# Protozoa-Derived Extracellular Vesicles on Intercellular Communication with Special Emphasis on *Giardia lamblia*

**DOI:** 10.3390/microorganisms10122422

**Published:** 2022-12-07

**Authors:** Bárbara Ferreira, Ágata Lourenço, Maria do Céu Sousa

**Affiliations:** 1 CNC-Center for Neuroscience and Cell Biology, University of Coimbra, 3004-517 Coimbra, Portugal; 2 Faculty of Pharmacy, University of Coimbra, 3000-548 Coimbra, Portugal; 3 CIVG-Vasco da Gama Research Center, EUVG-Vasco da Gama University School, 3020-210 Coimbra, Portugal

**Keywords:** extracellular vesicles (EVs), protozoan, *Trichomonas*, *Entamoeba*, *Leishmania*, *Trypanosoma*, *Plasmodium*, *Toxoplasma*, *Cryptosporidium*, *G. lamblia*, giardiasis, microvesicles (MVs), exosomes, parasite–host interaction

## Abstract

Parasitic diseases are an important worldwide problem threatening human health and affect millions of people. Acute diarrhea, intestinal bleeding, malabsorption of nutrients and nutritional deficiency are some of the issues related to intestinal parasitic infections. Parasites are experts in subvert the host immune system through different kinds of mechanisms. There are evidences that extracellular vesicles (EVs) have an important role in dissemination of the disease and in modulating the host immune system. Released by almost all types of cells, these nanovesicles are a natural secretory product containing multiple components of interest. The EVs are classified as apoptotic bodies, microvesicles, exosomes, ectosomes, and microparticles, according to their physical characteristics, biochemical composition and cell of origin. Interestingly, EVs play an important role in intercellular communication between parasites as well as with the host cells. Concerning *Giardia lamblia*, it is known that this parasite release EVs during it life cycle that modulate the parasite growth and adherence as well the immune system of the host. Here we review the recently updates on protozoa EVs, with particular emphasis on the role of EVs released by the flagellate protozoa *G. lamblia* in cellular communication and its potential for future applications as vaccine, therapeutic agent, drug delivery system and as diagnostic or prognostic biomarker.

## 1. Introduction

Parasitic diseases remain an important worldwide problem threatening human and animal health, even after so many years of technological and pharmaceutical evolution [1]. Globally, intestinal parasitic infections affect up to 3.5 billion people and around 450 million people fall ill because of intestinal parasites [2]. As these diseases mostly occur in developing countries and poor regions, they are somewhat neglected, even though they burden many people and cause multiple deaths every year. 

Owing to environmental, geographical, social, and other factors, the distribution and prevalence of various species of intestinal parasites differ from region to region. Parasitic infections are more prevalent between children as compared with the general population. A high prevalence of intestinal parasitic infection was reported among school children in Sub Saharan African countries like Ethiopia (27.7–95%), Central Sudan (90%), Rwanda (50%), Tanzania (48.7%), and Burkina Faso (84.7%) [2,3].

In general, intestinal parasitic infections are manifested by symptoms such as malabsorption, acute diarrhea, intestinal bleeding, reduced growth rate, and impaired work capacity that lead to important health, economic, and social problems. The most prevalent intestinal parasites are *Giardia lamblia*, *Entamoeba* spp., and *Cryptosporidium* spp. [3,4].

Anti-parasitic drugs are an important tool to combat parasites. Despite this, nowadays, protozoa resistances are already an important concern [5]. In addition, owing to climatic changes, the life cycle of parasites and their distributions are continually changing [6], so we still have much more to understand about how the parasites adapt and manipulate the host environment, evade the immune system, and communicate with one another and with their hosts, as well as the mechanisms behind this.

*G*. *lamblia* (synonym *G*. *duodenalis* or *G*. *intestinalis*) is a flagellated protozoan responsible for a zoonotic disease named giardiasis. Thereby, it has a board range of hosts, including domestic and wild mammals. Eight genetically distinct assemblages of this protozoan are known: humans are affected by the zoonotic assemblages, A and B, while assemblages C to H are animal-specific assemblages [7].

The parasite has two development forms: the trophozoite, with capacity to colonize the intestine, and the cyst form, which is excreted in faeces and responsible for the transmission of the parasite. The cysts are extremely resistant in environment, namely in water, thus being one of the most common causes of waterborne outbreaks of diarrheal disease [7,8]. 

The incubation period is one to fourteen days after cysts’ ingestion. Indeed, when exposed to the acid conditions of the stomach, an excystation process starts in which one cyst originates two trophozoites, which will continuously multiply by binary fission. To infect the host, the trophozoite must be able to attach host cells. In order to do so, the trophozoite has a ventral disk with the ability to attach to the intestinal epithelium as a clasping or suction-like mechanism. The adherence is also supported by adhesins, such as the trypsin-activated *G*. *lamblia* lectin (taglin), responsible for the first contact, and *G*. *lamblia* adherence molecule-1 (GLAM-1), which supports the avidity of the parasite to the enterocyte [7,8].

Clinical presentation of giardiasis can vary from acute to chronic diarrhea. Diarrhea is the main clinical sign of giardiasis and can occur with or without intestinal malabsorption syndrome [8,9]. However, extra-intestinal and post-infectious gastrointestinal complications are often reported, even if the mechanisms underlying this process remain unknow [10,11,12]. Despite that the focus is mainly on symptomatic patients, the asymptomatic form of the disease cannot be forgotten; once they harbor the parasite, contributing to the spread of the parasitosis, they have an important role in the transmission of giardiasis.

Extracellular vesicles (EVs), like ectosomes, plasma membrane microvesicles (MVs), exosomes, microparticles, and apoptotic bodies, are produced and released by almost all cells. Indeed, protozoa parasites also release these nanovesicles for extracellular milieu, including *G*. *lamblia*.

EVs represent a recent area of cellular biology in which the biogenesis, biochemical content, transport in the cells, and signal transduction are under intense investigation. Indeed, it seems that EVs play a crucial function in intercellular communication between parasite to parasite and parasite to host. However, its role in parasite infectiveness and the development of infection is still poorly understood [13].

The present work aims to review the role of EVs derived from protozoa, in parasite–host interaction, particularly on *Giardia*.

## 2. Extracellular Vesicles 

EVs are nanovesicles enclosed by a lipid bilayer and are released by prokaryotic and eukaryotic cells (normal and diseased cells), containing proteins, DNA, RNA, non-coding RNA, lipids, and metabolites [14]. These biomolecules allow communication with cells in the neighborhood, inducing, for example, tumor progression, immunotolerance, invasion, angiogenesis, and metastasis. They are expected to be found in bodily fluids in high concentrations related to cancer, infectious disease, or acute and chronic inflammatory diseases like atherosclerosis or diabetes mellitus [15,16,17,18]. 

As stated by the first International Society of Extracellular Vesicles in 2012, EVs were gathered into three different groups according to their releasing pathways, size, and sedimentation ability. Apoptotic bodies were considered all particles sized greater than 2 µm and pelleted at 2000–10,000 g, a very heterogenous group. Microvesicles ranged from 100 nm to 1 µm and pelleted at 10,000–20,000 g. The smallest particles are the exosomes, which have a size smaller than 100 nm and pelleted at >10,000 g [1,15,19,20]. 

Vesicle isolation methods include differential centrifugation, density gradients, precipitation, filtration, immunoisolation, and size exclusion chromatography [21,22]. 

The specific content of each EV is dependent on its biogenesis and on the cell type from which the EVs are secreted, and is influenced by the physiological or pathological state of the cell [23,24]. 

Because the content is so varied, distinction by its size is not enough and, for that reason, it is highly important to accurately characterize it in order to understand which nanovesicle type was isolated. EV subtype characterization should be carried out according to the physical characteristics (size or density), biochemical composition, experimental conditions inducing EVs’ secretion, and cell of origin. Nanoscale techniques, electron microscopy, Western blot, sulphosphovanilin assay, bead-based flow cytometry, mass spectrometry, and proteomics are examples of techniques that are commonly used for this purpose [21,22]. 

More recently, shedding from the plasma membrane of some tumour cells’ oncosomes, with a size up to 10 µm, has been found. It is relevant to emphasize that there is scientific evidence that the releasing of these vesicles may not be limited to tumour cells. These types of EVs cannot be distinguished from the other EVs by their size, but rather by their cargo. Moreover, these EVs represent populations of vesicles considered tissue-specific EVs, while apoptotic bodies, MVs, and exosomes are cell-derived EVs. The discovery of these particles indicates that, probably, the family of extracellular vesicles will always continue to increase [17,25]. 

The apoptotic bodies are formed during the programmed cell death (apoptosis). During the apoptosis, the cell condensates and the cytoskeleton collapses, the nuclear envelope disassembles, and the chromatin condensates. Altogether, these events result in cell fragmentation, where some membrane-enclosed fragments are formed, designated apoptotic bodies [15].

Exosomes are released by exocytosis through a secretory pathway with endosomal origin. Indeed, in the intracellular endosome, proteins, lipids, and other components are collected in multivesicular bodies (MVBs), which produce intraluminal vesicles (ILVs), afterward called exosomes, which mature and fuse with the plasma membrane [26]. 

Exosome production and release is dependent on at least three pathways that comprise the Endosomal Sorting Complex Responsible for Transport (ESCRT) [27], a ceramide-dependent mechanism [28], and the presence of tetraspanins [29,30].

ESCRT comprises four multiprotein complexes that assemble sequentially in the cytosol. These four multiprotein complexes are targeted to the endosome membrane through interaction with ubiquitin and/or clathrin [31,32]. The ESCRT-0, ESCRT-I, and ESCRT-II complexes have the ability to recognize and bind to ubiquitin, while the ESCRT-III complex is responsible for the final steps of the ILVs’ production process. Before the release of ILVs from the endosome membrane, ubiquitin is removed by an enzyme called de-ubiquitinase and, later on, ESCRT complexes are broken down by AAA-adenosine triphosphatase Vps4. Finally, through the action of a curvature-inducing factor, the ILVs are released. 

In the absence of ceramide, which is synthesized by the neutral sphingomyelinase 2, deformations in the endosome membrane occur and, consequently, the formation of ILV is compromised, thus interfering with the synthesis and release of exosomes. 

The presence of tetraspanins, transmembrane proteins that communicate with several signaling proteins, also influences exosome release. 

On the contrary, microvesicles come from a calcium-dependent mechanism and are secreted by the plasma membrane. This mechanism appears to be an innate property of most cells based on the enlarging of calcium in the cytoplasm. When a stimulus or cell damage leads to the increase in calcium, a mechanism activates the cleavage of the actin cytoskeleton by calpain mediator. Then, the proteins responsible for the movements of the lipids in the membrane, flippase and floppase, are inhibited and an ATP independent protein responsible for the distribution of lipids in the membrane is activated, the scramblase. This process results in the secretion of the MVs with phosphatidylserine exposure [15,16,33].

## 3. Protozoa EVs in Intercellular Communication

In the last decades, the role and potential of EVs of parasitic protozoa have aroused interest among researchers too (Table 1).

Regarding the protozoa parasite–host interaction, EVs can be divided into two groups: one released from the parasite and others released from the host cells. For instance, parasites like *G. lamblia* and *Trichomonas vaginalis*, which are extracellular parasites, secrete EVs; human cells that are infected by intracellular parasites, like *Leishmania* spp. or *Plasmodium falciparum*, also secret EVs [15,20].

The EVs have at least three ways of communicating with the recipient cells: (i) they can remain attached to the cell membrane, triggering signaling pathways owing to interaction with the cell receptor; (ii) they can be internalized by receptor-mediated endocytosis; or (iii) they can fuse with the membrane of the recipient cell [13,67].

### 3.1. Trichomonas vaginalis

*T. vaginalis* EVs are made up of RNA, cytoskeletal proteins, membrane proteins, and proteases that are also found in EVs released by other cells, but also have parasite-specific proteins such as BspA-like proteins and homologues of virulence proteins characterized in *Leishmania*, GP63-like metalloproteases, which seems to be involved in the pathogenicity of trichomoniasis [34,35].

Initial studies on proteome composition of the surface of *T. vaginalis* disclosed the presence of at least three tetraspanins (Tsps) [68,69]. Tetraspanins, present in mammalian cells, are involved in a wide variety of activities such as adhesion, fusion, motility, migration, and proliferation and are often used as markers of exosomes [34]. Thus, it is possible that *T. vaginalis* produces exosomes with Tsps in cargo participating in adherence and colonization of the urogenital tissue. The proteome analysis of exosomes isolated from the growth media confirmed the presence of parasite Tsps as well as proteins related to pathology [34,35]. Moreover, a component of the ESCRT complex, ESCRT-III subunit vacuolar protein sorting-associated protein 32 (VPS32), was identified in exosomes and MVs. Recently, it was demonstrated that VPS32 have an important role in biogenesis and cargo sorting of EVs and in the modulation of *T. vaginalis* adherence [70].

Adhesion of *T. vaginalis* to epithelial cells is critical for host colonization and establishment of infection [35]. When fusing with host cells, *T. vaginalis* EVs activate the expression of the surface receptor of epithelial cells, promoting the adhesion of the parasite (Table 1). Remarkably, incubation of epithelial cells with exosomes from a highly adherent strain increased the attachment of a less adherent strain [34]. Additionally, they modulate the expression of interleukins (ILs), increasing IL-6 expression and decreasing the IL-8 response [36]. The IL-8 is involved in the recruitment of neutrophils to the site of infection, thus its decrease allows the establishment of a chronic infection, while IL-6 is a mediator of chronic inflammation, having a pro-inflammatory effect [34]. Nievas and collaborators reported the release of microvesicles and large vesicles (LVs) by *T*. *vaginalis* in the presence of HeLa cells, consistent with a role of parasite EVs in modulating cell interactions [35]. Internalization of *T. vaginalis* EVs seems to occur via caveolar and lipid raft dependent endocytosis [71].

The local lesions caused by the parasite, as well as the fact that it is transmitted sexually, increase the risk of transmission of human immunodeficiency virus (HIV) or other sexually transmitted diseases, as well as the prevalence, progression, and severity of cervical and prostate cancer [36,71]. *T. vaginalis* can harbor an endosymbiotic RNA virus, the *Trichomonas* virus (TVV). New research found that TVV-positive *T. vaginalis* alter the cargo of secreted EVs and, subsequently, the host immune response is altered as well [72]. Proteome analysis of EVs released by TVV-positive *T. vaginalis* showed that TVV is present on the vesicles and these EVs induced a higher pro-inflammatory response in HaCaT cells compared with the TVV negative *T. vaginalis* EVs [73].

### 3.2. Leishmania spp.

Concerning EVs of the zoonotic protozoa, *Leishmania* spp., Silverman and collaborators reported the release of exosome from promastigotes forms of *L. donovani*, *L. major*, and *L. mexicana,* suggesting its participation in pathogenesis [37].

Like other parasites, *Leishmania* EVs can strongly influence macrophage cell function and signaling, demonstrating a pro-inflammatory effect, and have the capacity to attract neutrophils, exacerbating the resulting pathology [37,38,39,74]. Macrophages treated with derived exosomes of *L. donovani* produce IL-8, a pro-inflammatory cytokine [37]. Contrarily, the same authors showed that exosomes released by *L. donovani* have an immunosuppressive effect in dendritic cells, by stimulating IL-10 production and inhibiting and modulating TNF- α and IFN-γ, which enhance parasite progression [40].

The cases of exosomal presence of *Leishmania* GP63, elongation factor-1α (EF-1α), and chaperonin 10 (CPN10) proteins were reported as negative macrophage modulators [75,76]. GP63 is a protease that is present on the surface of the promastigotes and amastigotes forms and is one of the most important virulence factors because it allows the protection of the parasite from the lysosomal macrophage enzymes and allows the degradation of the extracellular matrix components, facilitating the migration of parasite out of the cells. When delivered to hepatocytes, this virulence factor was reported as responsible for the down-regulation of specific host miRNAs, promoting liver infection [77,78]. 

Mice studies on the role of derived *Leishmania* EVs are quite promising in the use of these nanovesicles in immunization programs. In fact, pro-inflammatory cytokines/ chemokines such as IL-12, IL-1β, and TNF-α are increased after exposure to EVs (Table 1) [41,42].

Despite the release of exosomes in vertebrate hosts, Atayde and collaborators reported the secretion of *L. infantum* and *L. major* exosomes in the midgut of sandflies by a mechanism resembling one found in mammals. During the biting of sandflies, in addition to promastigote forms, exosomes are also inoculated, probably increasing pathogenicity, mainly in cutaneous leishmaniasis [43].

### 3.3. Trypanosoma spp.

*Trypanosoma brucei* and *Trypanosoma cruzi* are the causal agents of sleeping sickness and the Chagas’ disease, respectively. Mice inoculated with EVs derived from trypomastigote forms of *T. cruzi* lead to an increase in IL-10 and IL-4 secretion (Table 1). EVs secreted by epimastigote and metacyclic forms (development stages in the *Triatominae* vector) contain glycoproteins, proteases (as cruzipain), cytoskeleton proteins, mucins, and associated GPI (glycosylphospatidylinositol)-anchored molecules that are critical for virulence and pathogenesis [44,79]. Cruzipain is present in trypomastigote, amastigote, and epimastigote forms of *T. cruzi* and is responsible for the degradation of host tissues, favoring immune evasion [80]. 

Beyond these proteins, the presence of small RNA in derived *T. cruzi* EVs has been reported, including tRNA [45]. More recently, studies with both parasite and infected host-cell-derived EVs revealed that the vesicles modulate the immune response, both *in vitro* and *in vivo* [46,47,48,81].

EVs isolated from blood of chronic patients present a different cargo compared with EVs from culture medium. Despite that more studies are still needed, the use of these nanovesicles as a biomarker can be a promising tool for control of the disease [82,83]. 

*T. brucei* EVs have several functions: (i) mediate the secretion of proteins, including proteases, crucial for the pathological process and the supply of nutrients [84]; (ii) modulate rRNA, snoRNA, and mRNA processing; (iii) mediate parasite–parasite communication, allowing the survival of the parasite on the vector, i.e., coordinate the pro-cyclic form during its migration from the midgut to the salivary glands of the tsetse fly, repelling it from less favorable environments [85]; and (iv) modulate parasite–host interaction, leading, for example, to EVs fusing with erythrocytes, causing their lysis and inducing anemia [49] and, on the other hand, modulate the host immune response through stimulation of macrophages and T lymphocytes [50].

### 3.4. Entamoeba histolytica

*E. histolytica*, the causal agent of amebiasis, is an intestinal protozoan of concern, especially in developing countries [86].

Recently, the identification and characterization of EVs released by this protozoan was reported [51,52]. *Entamoeba* EVs’ proteomes include elongation factors, heat shock protein 70 (HSP70), and ADP-ribosylation factor. Curiously, tetraspanin proteins were absent in these amebic EVs.

Amebic EVs seem to have a significant participation in encystation and in the modulation of human neutrophils (Table 1) [52]. Indeed, using EVs released by *E. invadens*, a model for amebic encystation, the Singh group remarked that these EVs promoted amebic encystation [51]. Together, these results suggest that EVs can play an important role in communication to surrounding parasites, ensuring the continuation of the biological cycle; stressful conditions induce encystation, while favorable conditions induce parasite replication. 

### 3.5. Plasmodium spp.

Malaria is an acute febrile illness caused by five species of blood protozoa parasites belonging to the *Plasmodium* genus: *P. falciparum*, *P. knowlesi*, *P. vivax*, *P. malariae*, and *P. ovale*. Malaria causes high levels of morbidity and mortality in human beings worldwide.

Exosomes retrieved from peripheral blood of infected patients with *P. vivax* and *P. falciparum* were widely reported. Moreover, clinical presentations of the disease, such as fever and cerebral dysfunctions, revealed different levels of circulating EVs, suggesting a significant role of EVs in the manifestation of disease [87,88,89]. Exosomes derived from *P. yoelii*-infected reticulocytes contained host and parasite proteins and modulate the immune response (Table 1). These data described for the first time that exosomes derived from reticulocytes could be investigated as a vaccine for malaria prophylaxis [53]. 

Several *in vitro* studies have been conducted to evaluate the role of *P. falciparum*-infected red blood cells’ EVs as mediators in both parasite–parasite and parasite–host communication and in modulation of the host immune response [54,55,56,57,58]. These EVs alter vascular function via regulatory Ago2-miRNA complexes and induce transcriptional changes in human monocytes [57,58].

### 3.6. Toxoplasma gondii

The protozoan *Toxoplasma gondii* causes toxoplasmosis and can infect any nucleated cell of humans and warm-blooded animals. During pregnancy, *T. gondii* is even more worrying because, if a pregnant woman became infected for the first time, abort and congenital malformations of the fetus can occur [59].

Early studies on the utilization of exosomes derived from *T. gondii*-infected cells, namely dendritic cells [59,60], macrophages [90], and foreskin fibroblasts [91], induced a pro-inflammatory immune response both *in vitro* and *in vivo*.

Furthermore, the effects of derived *T. gondii* EVs on macrophages [61,64] and mice [62] induce immune protection, with secretion of IL-10, IL-12, TNF-α, and iNOS, and increased the survival time of animals. Additionally, a recent investigation of Maia and colleagues in mice inoculated with *T. gondii* exosomes and microvesicles demonstrated the development of an immune response, in agreement with the previous reports, with high secretion of IgG1, IFN-γ, IL-10, and TNF-α (Table 1) [63].

### 3.7. Cryptosporidium parvum

*Cryptosporidium parvum,* an obligate intracellular protozoan, causes the diarrheal disease cryptosporidiosis that affects both human and animals [92]. The parasite is one of the most common causes of childhood diarrhea worldwide, with detrimental effects on the development of children. Moreover, it can also be life threatening to HIV/AIDS patients and transplant recipients.

After *C*. *parvum* infection, the epithelial cells increase the secretion of exosomes through the activation of TLR4/IKK signaling. Moreover, when the exosomes are placed in contact with *C*. *parvum* sporozoites, an anti-parasitic effect was reported [65]. Afterward, it was observed that exosomes released from *C*. *parvum*-infected epithelial cells induced an inflammatory response in primary splenocytes. Indeed, exosomes were able to stimulate a host immune response [66].

## 4. *Giardia* Extracellular Vesicles

The pathophysiological mechanisms of *Giardia* infection are multi-factorial and involve host and parasite factors, as well as immunological and non-immunological mucosal processes. Giardiasis causes intestinal barrier dysfunction via epithelial apoptosis and disruption of apical junctional complexes [93]. The tight attachment between *G. lamblia* trophozoites and intestinal epithelial cells through its adhesive disk reduces the small intestinal absorptive surface area and induces a diffuse shortening of the epithelial microvilli, causing disaccharidase deficiencies and malabsorption of nutrients, water, and electrolytes [94,95,96]. Moreover, the loss in intestinal brush border surface area, disaccharidase impairment, and increased crypt/villus ratios in giardiasis are mediated by activation of CD8+ T lymphocytes of the host via parasite secretory/excretory products [96]. 

The protozoan *G. lamblia* has undergone a reductive evolution and, therefore, has an elementary endomembrane system compared with other parasites, as it does not have an endosomal/lysosomal system, Golgi complex, peroxisomes, and mitochondria [97]. Alternatively, this parasite has peripheral vacuoles (PVs) that play the role of endosome and lysosome simultaneously, which are specialized endocytic cell components [98]. Moreover, *G. lamblia* also contains specific encystation secretory vesicles (ESVs) that have similar characteristics to the Golgi complex and have the function of controlling the release of constituents from the cyst wall. As *Giardia* does not have a Golgi complex, the release and transport of proteins occur directly from the endoplasmic reticulum to the plasma membrane or to cellular components, such as PVs. 

Like the other protozoa, *Giardia* is capable of shedding EVs, which participate in cellular communication and could modulate the pathophysiology of giardiasis and the immunity response of the host (Table 2) [99,100,101].

Evans-Osses and collaborators demonstrated that *G. lamblia* trophozoites are able to release MVs in response to different pH levels and calcium (vesicle inducer). They proceeded to electron microscopy study that revealed EVs with size between 60 and 150 nm and to nanoparticle tracking analysis that showed a peak with a mean diameter of 201.6 nm, corresponding to larger vesicles than exosomes. These authors also demonstrated the involvement of cholesterol in MV release, by observing an inhibition of MV production using different concentrations of methyl-β-cyclodextrin (MβCD). Furthermore, the absence of cholesterol inhibited the parasites’ attachment to the host cell, which was subsequently restored by the exogenous presence of MVs [101]. 

The pathogenic effect of trophozoites has been correlated with the secretion of proteins by parasite trophozoites during *Giardia*–host cell interactions. When *Giardia* secretome (secreted proteins) was placed in contact with IECs, alteration of IEC gene expression, cell signaling, and the production of pro-inflammatory cytokines were observed [102]. These findings suggest that one of the mechanisms by which *G*. *lamblia* secretes and transports proteins involves EVs.

**Table 2:** Role of *G. lamblia* extracellular vesicles in host cells.

EVs’ Source	Type	Main Cargo	Actions	References
Culture medium of *G.lamblia* trophozoites, isolate WB clone C6	Microvesicles	α-tubulin; ADI; β-tubulin; Enolase; Giardin; HSP70; OCT; Ribossomal protein; rRNA; VSPs; ZFD protein	Increase trophozoite adhesion to Caco-2 cells	[101]
			Activation and allostimulation of human DCs	
Culture medium of *G.lamblia* trophozoites, isolate WB clone C6	Large and small EVs	ADI; α-1 giardin; Ankyrin; Arginine-metabolizing enzymes; Cathepsin B; FixW protein; Giardins; Katanin; OCT; Peroxiredoxin-1; PFOR; VSPs	Increase trophozoite attachment to Caco-2 cells (only large EVs)	[103]
Culture medium of *G.lamblia* trophozoites, isolate NF	Extracellular vesicles	Cathepsin B cysteine proteases; VSPs; ADI; Tenascins; Tubulin β-chain; Pyruvate phosphate dikinase; PFOR; EF-α; HSP90; OCT; Tubulin α- chain; NADH oxidase; EF-γ; Phosphoglycerate kinase; EF-2; Cluster of kinase activity; NADH oxidase; Peroxiredoxins	Disruption of tight junctional proteins, Zonula occludins-1, and claudin-4 of SCBN cells;	[104]
			Antibacterial effect on *Escherichia coli* (HB101 strain) and *Enterobacter cloacae*	
Culture medium of *G. lamblia* trophozoites, isolate WB clone C6	Extracellular vesicles	14-3-3 proteins; ADI; α-tubulin; Giardin; HSPs; OCT; VSPs	Proinflammatory response in murine macrophages	[105]
			Activation of TLR2 and NLRP3 inflammasome signaling pathways	
Culture medium of *G.lamblia* trophozoites, isolate WB clone C6	Extracellular vesicles	NA	Activation of the p38, ERK, and NF-κB signaling proinflammatory pathways in murine macrophages	[106]
Culture medium of *G.lamblia* trophozoites, isolate WB clone C6	Extracellular vesicles	NA	Therapeutic effects on experimental murine colitis	[107]
Culture medium of *G.lamblia* trophozoites, isolate WB clone C6	Large and small EVs	Alteration of proteome profile by nystatin and oseltamivir treatment	Decrease in attachment and encystation	[108]

ADI: arginine deaminase; HSP: cytosolic heat shock protein; OCT: ornithine carbamoyl; rRNA: ribosomal RNA; VSPs: variant surface proteins; ZFD protein: zinc finger domain protein; DC: dendritic cell; EVs: extracellular vesicles; PFOR: pyruvate-ferredoxin oxidoreductase; TLR2: toll-like receptor 2; NLRP3: NOD-, LRR-, and pyrin domain-containing protein 3; EF: elongation factor; NA: not applicable; ERK: extracellular-signal-regulated kinase; NF-κB: nuclear factor kappa B.

**Figure 1:**
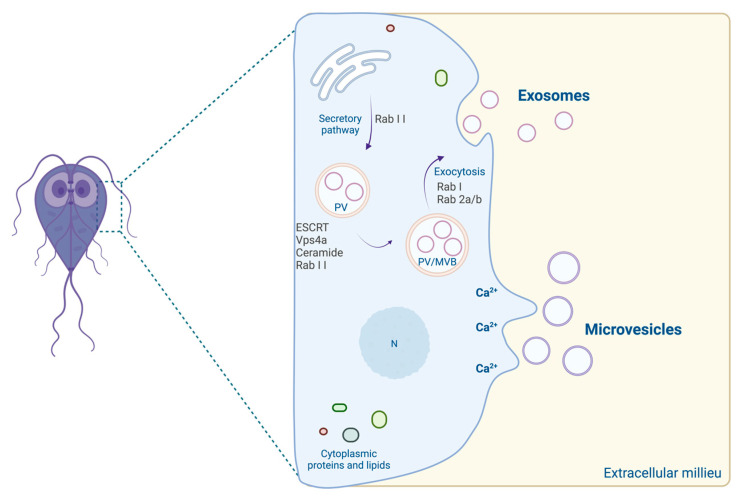
Proposal biogenesis of *Giardia* EVs. (PV: peripheral vacuoles; ESCRT: endosomal sorting complexes required for transport; MVB: multivesicular bodies; N: nucleus) (created with BioRender.com).

*G. lamblia* has a reduced ESCRT, lacks tetraspanins, and is unable to synthesize ceramide *de novo* [109]. This parasite appears to have a unique mechanism of exosome-like vesicle formation. Studies suggest that exosomes’ biogenesis occurs in PVs and that their production and release is dependent on ESCRT-associated protein Vps4a, Rab 11, and ceramide. The Rab 11 protein participates in cell differentiation and division and seems to be involved in the communication between the ER and the VPs. Rab 1 and Rab 2 a/b proteins, related to exocytic vesicular trafficking, appear to be involved in exosome exocytosis (Figure 1).

A subsequent study of Gavinho and collaborators described two distinct EV populations from *G. lamblia* (with different protein contents), where large EVs, but not small EVs, are associated with effective parasite adhesion to the host. They suggest that the two EV populations identified in *G. lamblia* so far, large and small EVs, have distinct functions in the phenotype of this pathogen and can be selectively modulated using peptidylarginine deiminase (PAD) inhibitor and cannabidiol (CBD). As the attachment to the epithelial intestine is fundamental, and large EVs clearly aid this process, the use of selective EV inhibitors, such as clamidine, can be used to interfere with EV secretion, blocking the *Giardia* life cycle [103]. 

Notably, a bacteriostatic effect was observed when *Giardia* EVs were exposed to commensal gut bacteria [104].

Proteomic analysis of G. lamblia EVs revealed the presence of important proteins to guarantee the survival of the parasite and to regulate the infection. The main proteins identified in EVs cargo include variant surface proteins (VSPs), cytosolic heat shock protein 70 (HSP70), β-tubulin, α tubulin, giardin, arginine deaminase (ADI), ornithine carbamoyl transferase (OCT), enolase, and cysteine proteases [101,103]. Some of them are *Giardia* antigenic proteins, such as the VSPs, the excretory-secretory products (ESPs), the annexin homolog α1-giardin, α11-giardin, α-enolase, and OCT [110,111,112,113]. VSPs are rich in cysteine proteins and play an important role in the coverage of trophozoite, which will create the perfect conditions to evade the immune system. The antigenic variation is controlled by the interference RNA, which leads to the expression of different VSPs [102,114]. The HSP70 is important to the folding and unfolding of proteins, permits the survival of the cell under stress conditions, and stimulates the immune system. The HSP70 has been described in association with encystation-specific secretory vesicles (ESVs). Giardin is involved in the attachment of parasite to the microvilli of the epithelium and induces the production of anti-*Giardia* antibodies (IgA and IgG), creating protection against a new infection [115]. The ADI and OCT are part of a strategy to escape to the nitric oxide (NO) defence mechanism, competing for the same substrate for the production of NO [102]. The Enolase is an enzyme that participates in the glycolysis pathway and is crucial for parasite survival and virulence [115]. Cysteine proteases, like cathepsin B-like protein, disrupt the intestinal epithelial barrier; degrade inflammatory chemokines, such as IL-8, that will lead to the decrease in neutrophils; and attenuate inflammation [102,116,117]. 

In order to address how *Giardia* EVs could modulate the host cell innate immunity, Zhao and colleagues investigate its effects in mouse macrophages and reported the secretion of inflammatory cytokines IL-1β, IL-6, IL-10, IL-12, IL-17, IFN-γ, TNF-α, IL-18, CCL20, and CXCL2 and the activation of TLR2 and NLRP3 inflammasome signaling pathways [105]. Furthermore, the activation of the p38, ERK, and NF-κB signaling pro-inflammatory pathways has also been reported, evidencing once again that *Giardia* EVs trigger an innate immune response on host cells [106]. Interestingly, in a mouse model of colitis, the treatment with EVs decreased pro-inflammatory cytokines (IL-1β, IFN-γ, and TNF-α) [107]. Observation of contradictory effects of EVs secreted by the same parasite is quite frequent and seems to be related to stimulus-dependent EVs’ production and the isolation methods used [22]. 

Our team work also demonstrated that *Giardia* EVs evoke a pro-inflammatory profile on macrophages cells and DCs and induce adaptive immune response in mice (patent PCT/IB2022/057466; ref. P1303-7WO) [118]. 

**Figure 2:**
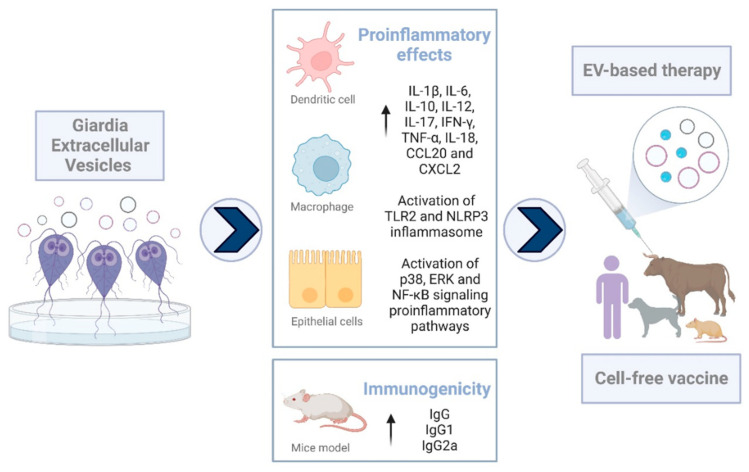
Effects of *Giardia* EVs in host cells (IL: interleukin; IFN-y: interferon-gamma; TNF-α: tumor necrosis factor α; EV: extracellular vesicle; TLR2: toll-like receptor 2; NLRP3: NLR family pyrin domain containing 3; ERK: extracellular signal-regulated kinase; NF-κB: nuclear factor kappa B) (created with BioRender.com).

According to the data published, we summarize the effects of *Giardia* EVs in host cells in Figure 2, emphasizing the potential application of these EVs in a new therapeutic approach and vaccine development.

### EVs: Future Applications

The potential role of EVs in a large number of biological processes, jointly with many of their fascinating characteristics, forms the basis of extending EVs research.

The use of EVs as a disease biomarker is very promising, whether for diagnosis or prognosis. For example, the qualitative analysis of EVs derived from blood of chronic Chagas disease patients revealed the presence of *T. cruzi* antigens in the vesicles [83].

As EVs are a pathway for exchanging information between cells, they can be a potential target for several therapeutic approaches [119]. Studies suggest that, in the absence of cholesterol, *Giardia* EVs are not released and the parasite adherence to the intestinal cells of the host is compromised. Moreover, the treatment of *Giardia* with nystatin and oseltamivir, two inhibitors of lipids rafts, induces an alteration in the size and the proteome profile of the secreted *G. lamblia* EVs [108]. Furthermore, new research showed that *T. brucei*-derived EVs promote erythrophagocytosis, causing anemia during acute trypanosomiasis [49], and that *P. falciparum* EVs have an important role in the development of artemisinin-resistant malaria parasites [120]. Therefore, blocking the synthesis and release of EVs, or altering the EVs cargo to prevent the survival of the parasite in the host, represent potential therapeutic strategies for protozoa parasites.

The application of EVs to generate immunization via a vaccine can be the next step, as the connection between the vesicle and cells from the immune system appears to be more efficient in creating a T-cell response. In order to do so, the study of DC-derived EVs would be interesting, as DCs are able to secret EVs with MHC II antigens and produce an adaptive response. This way, EVs will function as vehicles and enhancers of the immunogenicity of antigens. Studies like these have already been reported, with *Plasmodium yoelli* [53], *Toxoplasma gondii* [59], and *G. lamblia* [105]. 

As reviewed above, EVs are associated with multiple effects: improve the attachment of parasites to target cells, increasing pathogeny; stimulate and modulate host cells, inducing an anti- or pro-inflammatory response; help parasites evade the host’s immune response; and alter gene expression in recipient cells. Taken together, all of these findings make EVs an excellent tool in the fight against protozoa diseases and a target for future applications as a therapeutic agent, as a vaccine antigen, and as a biomarker of diagnosis or prognosis of protozoosis, namely in giardiasis.

## 5. Conclusions

In this review, we extensively described the effects of protozoa EVs (pro- and anti-inflammatory) and the mechanisms behind it. Undoubtedly, the protozoa-derived EVs have a crucial role in intercellular communication including with other parasites, microorganisms, and host cells.

Despite all of the efforts that have been made for parasitic diseases’ control, parasitosis is still an unmet clinical problem and EV-based therapy seems to bring us one step closer to a possible solution. 

However, compared with the knowledge of mammalian EVs’ biogenesis, we still have a gap in our understanding regarding EVs’ biogenesis of flagellate and non-flagellate protozoa. 

The techniques used in the production and characterization of protozoa EVs are still a concern regarding the reproducibility of results. Efforts must be made to characterize EVs using several cellular and molecular markers. 

## Figures and Tables

**Table 1:** Role of protozoa extracellular vesicles in host cells.

Disease	EVs’ Source	Type	Actions
Phylum Sarcomastigophora			
Trichomoniasis	Trophozoite of *T. vaginalis*	Exosomes	Modulate host immune response and increase parasite attachment [34]
		Microvesicle-like structures and large vesicles	Modulate cell interactions [35]
		Exosome like vesicles	Increase IL-10, IL-6, and TNF- α secretion in mouse macrophages; Increase IL-10 and decrease of IL-17 production in infected murine model [36]
Leishmaniasis	Promastigotes of *L. donovani*, *L. major,* and *L. mexicana*	Exosomes	Delivery of proteins in mouse macrophages and increase of IL-8 [37]
	Promastigotes of *L. amazonensis*	Extracellular vesicles	Stimulate bone-marrow-derived macrophages and peritoneal B-1 cells [38]
	Promastigotes of *L. amazonensis*, *L. braziliensis*, and *L. infantum*	Extracellular vesicles	Activation of macrophage immunomodulatory response (only EVs of *L. amazonensis*) [39]
	Promastigotes of *L. donovani*	Exosomes	Immunosuppressive effect [40]
	*L. amazonensis*-infected mouse macrophages	Extracellular vesicles	Increase in IL-12, IL-1β, and TNF-α [41]
	Long-term and in-vivo-derived promastigotes of *L.amazonensis*	Extracellular vesicles	Partial protective effect in an immunization model [42]
	Lavages of midgut of sandflies-infected *L. infantum* and *L. major*	Exosomes	Participation in pathogenesis and modulate monocyte cytokine response [43]
Chagas Disease	Trypomastigotes	Extracellular vesicles	Increase tissue parasitism and inflammation; Increase in IL-10 and IL-4 secretion [44]
		Extracellular vesicles	Promotes metacyclogenesis [45]
		Extracellular vesicles	Activation of macrophages Production of TNF-α by dendritic cells (mice) [46]
	Infected human macrophages	Extracellular vesicles	Interact with TLR2 and stimulate the translocation of NF-κB [47]
	Plasma of chronic patients	Extracellular vesicles	Increase in IFN-y and decrease in IL-17 production [48]
African Trypanosomiasis	Trypomastigotes	Extracellular vesicles	Mediate transfer of virulence factor and induce anemia [49]
		Extracellular vesicles	Induce differentiation of M1- and M2- macrophages; Generation of MHCI+, MHCII+ and MHCI+, MHCII+ macrophages; Expression of CD3 and nuclear factor FoxP3 [50]
Amebiasis	Encysting parasites	Extracellular vesicles	Promoted encystation [51]
	*E. histolytica* trophozoites	Extracellular vesicles	Immunomodulatory effects on macrophages [52]
Phylum Apicomplexa			
Malaria	*P. yoelii*-infected reticulocytes	Exosomes	Induce immune protection [53]
	*P. falciparum*-infected erythrocyte	Exosomes-like vesicles	Delivery of genes [54]
		Microvesicles	Immunostimulatory and act as messengers between iRBCs [55]
		Extracellular vesicles	Alter vascular function [56]
		Extracellular vesicles	Modulate monocyte cytokine response [57]
		Extracellular vesicles	Decresase of parasite invasion [58]
Toxoplasmosis	Dendritic-cell-derived pulsed *ex vivo* with *T. gondii* antigens	Exosomes	Induce immune protection [59]
	Splenic dendritic cell pulsed *in vitro* with *T. gondii*-derived antigen	Exosomes	Induce immune protection [60]
	Tachyzoites	Exosomes/Microvesicles	Modulate macrophage activation; induce immune protection [61,62,63]
		Extracellular vesicles	Induce IL-10, TNF-α, and iNOS secretion [64]
Cryptosporidiosis	*C. parvum*-infected epithelial cells	Exosomes	Anti-parasitic activity [65]
	*C. parvum*-infected epithelial cells	Exosomes	Activate immune cells [66]

IL: interleukin; TNF: tumor necrosis factor; EVs: extracellular vesicles; MHC: major histocompatibility complex; TLR: toll-like receptor; NF-κB: nuclear factor kappa B; IFN-y: interferon-gamma; iRBCs: infected red blood cells; iNOS, inducible nitric oxide synthase.

## Data Availability

Not applicable.
